# Determination of Biogenic Amines in Fresh Fish and Processed Fish Products Using IC-MS/MS

**DOI:** 10.3390/foods10081746

**Published:** 2021-07-29

**Authors:** Drago Kočar, Sevim Köse, Bekir Tufan, Andrej Ščavničar, Matevž Pompe

**Affiliations:** 1 Faculty of Chemistry and Chemical Technology, University of Ljubljana, Večna pot 113, 1000 Ljubljana, Slovenia; andrej.scavnicar@fkkt.uni-lj.si (A.Š.); matevz.pompe@fkkt.uni-lj.si (M.P.); 2 Faculty of Marine Sciences, Karadeniz Technical University, Camburnu, 61530 Trabzon, Turkey; kosesevim@gmail.com (S.K.); bekirtufan@gmail.com (B.T.)

**Keywords:** biogenic amines, determination, fish products, histamine, ion chromatography, MS/MS

## Abstract

A new method was proposed for the determination of underivatized biogenic amines based on ion-exchange chromatography coupled with mass spectrometry detection. The method was applied to the analysis of 10 biogenic amines in fresh and processed fish products. The amines were extracted from muscle tissue with water without any additional derivative step or sample clean-up. Separation of biogenic amines was done by the IonPac (4 × 50 mm) column, applying a gradient eluent by mixing formic acid (2 mol L^−1^) and Milli-Q water (formic acid concentration from 400 mM to 2 M). The results demonstrated a linear response in the range of 0.01 to 10 mg L^−1^. The detection limits for the fish products ranged from 20 ng/g up to around 400 ng/g for histamine and putrescine, respectively. Spermidine and spermine showed significantly higher detection limits. This current method can be used for the determination of biogenic amines in both fresh and processed fish products for regulatory purposes and monitoring food-safety issues relating to these amines, particularly histamine. It is also a useful method for evaluation of other commercial analytical test kits and commonly used methods that are possibly affected by the food matrix due to processing or other drawbacks arising from the derivatization process.

## 1. Introduction

The interest in the determination of biogenic amines (BAs) in fresh and processed foods is growing not only due to their toxicity, but also due to the levels of BAs in food products often being considered as a marker of spoilage during storage or ripening, and therefore, a quality index [1,2]. The consumption of foods containing high concentrations of BAs may cause some adverse effects to some consumers, such as headache, hypo- or hypertension, nausea, cardiac palpitation, renal intoxication, and in severe cases, intracerebral hemorrhage and death [3,4]. The formation of BAs in food depends mainly on the microbial decarboxylation of available free amino acids in the product. Therefore, the amount of BAs in the products varies and is closely related to the types of available free amino acids, the presence of BA forming bacteria, and the conditions for microbial activity such as suitable time/temperature, pH, and water activity. The most frequent kind of food intoxication relating to BAs is histamine poisoning, previously known as “scombroid fish poisoning” due to its first involvement being reported from *Scombridae* family such as tuna. The secondary amines such as putrescine (PUT) and cadaverine (CAD) also play an important role in food poisoning, as they can increase the toxicity of histamine (HIS) or react with nitrites to form carcinogenic nitrosamines.

The variability in dose/response for HIS toxicity to human health has been reported in relation to different factors, such as the presence of other BAs potentiating histamine food poisoning (HFP) [5,6]. Depending on the reported cases, a guideline was suggested for the HIS levels in fish as regards a health hazard. According to this guideline; <50 ppm HIS is regarded as safe for consumption, while the levels of 50–200 ppm and 200–1000 ppm HIS are suggested as possibly and probably toxic, respectively. The levels >1000 ppm HIS are accepted as toxic and unsafe for human consumption. However, in most cases, HIS levels with implicated fish have been above 200 ppm, often above 500 ppm [6,7]. Therefore, a hazardous HIS level of 500 ppm has commonly been suggested for most food authorities while lower levels of 50 ppm have also been reported in HFP [6,8]. Currently, no recommendations on the levels of other BAs, with exception of tyramine (TYR), have been suggested for human consumption. For adults, values of 100–800 ppm for dietary TYR and 30 ppm for dietary phenylethylamine (PHE) have been reported as toxic doses in foods. The ingestion of 60 ppm of dietary TYR can cause migraine in individuals using monoamine oxidase inhibitor (MAOI) drugs, while 100–250 ppm will produce a hypertensive crisis [9]. However, only HIS was regulated by the government authorities in fish and fishery products. Controlling the presence of BAs, particularly HIS, is stricter for fish and fishery products than other food products around the world. The regulatory limits for HIS presence in fish products were imposed more than 20 years ago. According to the European Union Directive [10,11], nine samples must be taken from each batch of fishery product from fish species associated with a high amount of histidine, particularly fish species of the families *Engraulidae*, *Clupeidae*, *Coryfenidae*, *Pomatomidae*, *Scombridae,* and *Scombresosidae*. These samples must fulfill the following requirements.

The average HIS content must be 100 ppm or less, while no more than two samples may have levels between 100 ppm and 200 ppm, and no sample may have a level above 200 ppm. However, the EU regulations stipulated higher critical levels of HIS for “the fishery products which have undergone enzyme maturation treatment in brine and manufactured from fish species associated with a high amount of histidine” [11]. According to the regulation, the average HIS content must be 200 ppm or less, no more than two samples may have levels between 200 ppm and 400 ppm, and no sample may have a level above 400 ppm. For fish sauce, only one sample is to be taken, and HIS content should not exceed 400 ppm [11]. However, the FDA [8] set a stricter upper allowable limit for HIS as 50 ppm for fish species, since HIS is generally not uniformly distributed in decomposed fish, and numerous outbreaks were caused by this amount. If 50 ppm is found in one section, there is the possibility that other sections may exceed the toxicity level of 500 ppm [8]. Other countries in the world usually follow the rules of either the EU or FDA [6]. No regulations were made for other BAs in fish products.

Histamine food poisoning is now the most prevalent form of seafood-borne disease in the United States, as well as in other parts of the world [6,7,8,9,10,11,12,13,14,15,16]. Lehane and Olley [13] pointed out two main problems in controlling HFP. One is the lack of standardization of HIS detection methodology, with numerous different methods being used around the world; the second is that the presence of HIS alone is not necessarily a reliable indicator of fish likely to cause HFP, since it was already mentioned that some other amines can potentiate HIS toxicity. A very exhaustive overview on the role of BAs in intoxication, spoilage, and nitrosamine formation in fish was published by various authors [6,12,13,16,17,18]. In order to address all these problems, one needs a reliable analytical procedure for measuring all BAs in fish and fishery products.

Various methods are described in the literature for the determination of BAs or HIS only [6,12,17]. Analytical methods for BAs can be divided into two main groups in terms of interested parties, namely food regulators/food trade associations and food-processing companies. Food-processing or trade companies are particularly interested in fast, cheap, and user-friendly methods that can be routinely used for monitoring HIS formation in their production system. Suitable methods for such purposes are colorimetric, fluorometric, and immunoassay methods, or thin layer chromatography for semi-quantitative analysis. These methods generally measure only HIS. However, regulatory or trade establishments are interested in more advanced methods that would provide sensitive measurements for the simultaneous determination of HIS and other BAs. Although the AOAC fluorometric method is officially accepted as a sensitive method by the FDA for measuring HIS only, high-performance liquid chromatography (HPLC) methods were approved by most regulatory offices and reference laboratories for detecting BAs in fishery products [6,8,10,11,12,19,20]. However, these methods have the disadvantage of using derivatization steps, as well as various other drawbacks such as cumbersome sample preparation [6,17,20,21,22].

In order to avoid derivatization steps, liquid chromatography (LC) was coupled with pulsed amperometric detection (PAD) or integrated pulsed amperometric detection (IPAD) using noble-metal electrodes in alkaline media [23]. These techniques also have the disadvantage of requiring a post-column addition of a pH modifier. On the other hand, suppressed conductivity or mass spectrometry (MS) detection does not need any pre-column or post-column modification. The former technique was recently proposed for the determination of HIS in tuna [22]. The method was not tested for other types of fish products, and neither for whole types of important amines, other than four BAs.

A couple of methods exist for detecting and quantifying underivatized BAs using the LC-MS/MS approach [24,25,26]. Amongst these methods, Sagratini et al. [24] developed a solid-phase extraction method using HPLC/MS/MS detection to determine BAs in hake, and Self et al. [25] and Romero-Gonzalez et al. [26] reported analytical procedures for the determination of BAs in tuna using Hilic-UHPLC/HRMS detection and in anchovy using UHPLC/MS/MS detection. However, the method of Sagratini et al. [24] involves a sample clean-up procedure that is still a cumbersome application for routine analysis, and might cause amine loss. In addition, the method was applied to a fish species that is not in the list of species claimed to contain a high HIS health risk. None of the three studies have been used to determine all 10 BAs together that are important in fish and fishery products. Moreover, although the related methods showed good detection limits with reasonable recoveries for the tested amines in fish, only limited fish species were used for the mentioned studies. As the sample matrix, particularly for traditional fish products, varies to a great extent, an effect on the method performance is also expected, especially when excluding the derivatization step. Therefore, there is still a need to apply MS/MS detection in different sample matrices of fish and fishery products without any sample clean-up or derivatization step.

With regard to BAs, the main drawback of HPLC is unsatisfactory separation on reversed-phase (RP) columns. In addition, mobile phases used with HPLC (acetonitrile, methanol) often contain impurities that may have a negative effect on MS detection [27]. Replacing HPLC with ion-chromatographic separation alleviates the problems mentioned, especially since IC columns designed specifically for the separation of BAs are readily available on market. Few studies applied IC for the detection of BAs in foods and several drawbacks were also reported, such as lack of selectivity, which was discussed in detail in our earlier publication [28]. In our previous study, an IC-MS/MS method was applied to cheese products to determine 10 BAs, and obtained good separation. However, different food matrices can affect the method performance, including its recovery and sensitivity. Therefore, there is still a need to develop a rapid and sensitive method to determine BAs in fresh fish and processed fish products.

This paper describes a method that is capable of simultaneous determination of 10 BAs, namely agmatine (AGM), CAD, HIS, PUT, TYR, PHE, spermidine (SPMD), spermine (SPM), trimethylamine (TMA), and tryptamine (TRT). Using an ion-chromatographic approach instead allows for better separation, whereas MS/MS detection results in less interference, lower detection limits, and less labor-intensive sample preparation. It will also be shown that the use of IC/MS/MS is favorable in comparison to HPLC/MS/MS, since it provides better results in terms of separation, as well as simplified handling and sample pre-treatment.

## 2. Experimental Analysis

### 2.1. Materials and Chemicals

The agmatine sulphate (≥97%), cadaverine dihydrochloride (≥98%), histamine dihydrochloride (≥99%), 2-phenylethylamine (≥98%), putrescine dihydrochloride (≥98%), spermidine trihydrochloride (≥99%), trimethylamine hydrochloride (≥98%), tyramine hydrochloride (≥99%), and tryptamine hydrochloride (≥99%) were supplied by Sigma Aldrich, and the spermine tetrahydrochloride (≥99%) was purchased from Fluka (Buchs, Switzerland). The water used for the preparation of solutions and chromatographic separations was obtained from a Milli-Q water purification system (Millipore). All the other chemicals were provided by either Sigma Aldrich (Buchs, Switzerland) or Fluka. All the reagents used in this work were analytical grade.

The concentration of stock standard solution was 1000 mg L^−1^ of each amine, referred to as the free base. The stock standard solution was prepared in a 100 mM solution of formic acid and was stored in refrigerated conditions at 4 °C for further dilution. Diluted solutions were prepared weekly.

Fresh fish and processed fish products were provided from markets/supermarkets of both Slovenia and Turkey, although some products originated from 7 different countries. Fermented fish was bought directly from the producing company. The products were transferred to the laboratory under cold-storage conditions and kept either at 4 °C or −18 °C when necessary. For recovery and validation studies, brined herring, fresh and smoked salmon, fresh tuna, and fresh swordfish were used. For the application studies of real samples after method validation, five main types of traditional fish products were chosen to determine BA contents. These were mainly fermented fish, including fish paste, dry salting, brining, marinated, and smoked fish products. These products were produced from 5 different fish species (anchovy, bonito, herring, bluefish, and salmon). Despite some similarities, products within the same type varied according to commercial brands.

### 2.2. Extraction of the Samples

The products were taken out of their pack, and the flesh of the fish samples was separated from the skins and bones manually. The products in oil or brine were first taken out from their pack after draining their liquid or oil, then the remaining oil or liquid was further drained on a strainer and dried off with the help of a paper towel. The flesh of fish samples from each lot was collected in a food processor (Gorenje, MPE 700 EA, 700 W, Europe) and homogenized for 5 min at maximum speed. Then, 10 g of the sample from each lot was placed in a separate beaker, followed by the addition of 100 mL of Milli-Q water. Each sample was first mixed in an ultrasonic bath for 5 min, after which each mixture was transferred to a mixer to be further homogenized for 25 min. Each sample was filtered using Whatman 1 filter paper. The first 2–3 mL of filtrate was discarded. The supernatant was further filtered into a vial using a disposable syringe and Millex-LH filter unit (cat. no. SLLHRO4NL). After discarding the first 2–3 drops, the filtered extracts were analyzed immediately or stored in a plastic bottle at −18 °C until analysis.

The preparation of spiked samples was carried out in the same way, except that a known amount of stock standard solution of BAs was added to 10 g of the sample before the addition of water. After spiking, the samples were mixed well and kept in cold storage for 30 min. The remainder of the procedure was the same as described above. For extraction-recovery evaluation, 5 replicate homogenized samples were weighed (approx. 10 g) and a stock standard mixture of 10 amines (1 mg/mL of each) was added as follows: 0, 10, 20, 30, and 50 μL. The extractions into 100 mL of water followed, and the theoretical concentration of added analyte in fish were 0 ppm, 1 ppm, 2 ppm, 3 ppm, and 5 ppm, respectively.

### 2.3. IC-MS/MS Analysis

Ion chromatography analysis was carried out using an HPLC system by Perkin Elmer that consisted of a PE200 binary pump and a PE200 autosampler. The separation of BAs was performed on an IonPac CG 17 cation-exchange column (4 × 50 mm, Dionex, CA, USA) using a gradient elution procedure. The usual choice for mobile phase, non-volatile methanesulfonic acid, was replaced with a more volatile one to prevent the formation of deposits in the mass spectrometer’s ion source. Several other volatile organic acids were considered; namely, acetic acid, propionic acid, and haloorganic acids. However, with organic acids, the appropriate ionic strength to obtain good separation was not achieved. Haloorganic acids, on the other hand, act as ionization suppressants, leading to higher detection limits. Therefore, formic acid was the appropriate mobile phase that enabled appropriate separation conditions and non-suppressed mass spectrometric detection. The linear gradient was obtained using 2 eluents. These were formic acid with a concentration of 2 mol L^−1^ and Milli-Q water. The concentration of the formic acid in the final eluent varied from 400 mM up to 2 M. The exact gradient used is presented in Table 1. The analysis time was 50 min. An additional 5 min was allowed for system conditioning. Ion chromatography was performed without suppression. The flow rate and the injection volume were 1 mL min^−1^ and 25 μL, respectively.

The individual BAs were detected using a 3200 QTrap mass spectrometer (Applied Biosystems, MA, USA) in multiple-reaction-monitoring (MRM) mode. The ionization used was electrospray, and the following ion source experimental conditions were applied: temperature 400 °C, ion-spray voltage 5500 V, curtain gas setting 20 psi, ion-spray gas setting 20 psi, and auxiliary gas setting 60 psi. The conditions for MRM (i.e., fragmentation conditions and fragment intensities) were optimized for each analyte separately to obtain the highest possible sensitivity. MRM transitions are given in Table 1.

## 3. Results and Discussion

The main objective of this study was the development of a reliable ion-chromatographic method for the accurate determination of the main BAs present in fresh fish and processed fish products. Special attention was devoted to the determination of TRY, which is known to be incompatible with cation suppressors, and therefore is impossible to detect using standard conductometric detection. Short investigation determined the mechanism of TYR removal in the cation suppressor. The suppressor was used in chemically regenerated suppression mode: separate flows were pumped through the eluent channel and suppression channel. The eluent flow contained 10 μmol/L TYR solution. No TYR was detected in the eluent flow after the suppressor; however, it was detected in the regeneration flow. Therefore, we concluded that the TYR passed the suppressor membrane under its operating conditions. Due to these findings, the detection was performed on an MS/MS instrument, which enabled reliable detection of TYR as well. During the method development; that is, coupling of ion chromatography with an MS detector, one must consider the limitations of both techniques. The usual choice, non-volatile methanesulfonic acid, was replaced with a more volatile one in order to prevent the formation of solid deposits in the mass spectrometer. Several volatile organic acids (acetic and propionic acids) mentioned in Section 2 were applied without a successful separation, even at high concentrations. Other stronger organic acids, such as haloorganic acids, which are considered as potential candidates, are known to be volatile enough to be compatible with MS. However, their main drawback is that they may suppress ionization. For example, it is known that trifluoroacetic acid suppresses ionization in MS experiments, leading to higher detection limits. In the end, formic acid was chosen, which also enabled appropriate separation conditions and non-suppressed mass-spectrometric detection. Successful separation was also experienced in our earlier study in the determination of BAs in cheese samples [28].

**Figure 1:**
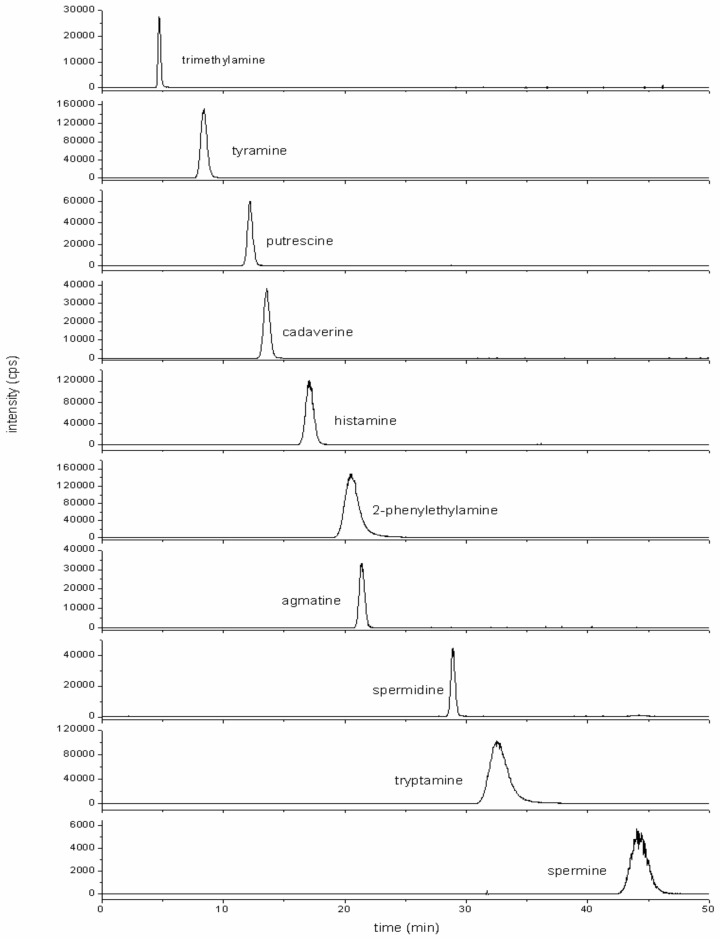
MRM chromatograms of standard mixture of biogenic amines. *Concentration of each of the amines was 10 mg L^−1^*.

The separation capability of the CS17 analytical cation-exchange column was also tested. Due to the strong retention of some BAs and lower elution power of formic acid compared with methanesulfonic acid, the retention times of some BAs were very long; that is, several hours, even though the concentration of formic acid in the eluent was raised up to 2.00 M. In order to lower retention times, a lower-capacity guard column was used for the separation. The results demonstrated a satisfactory separation with a shorter CG17 cation-exchange guard column. The separation of BAs was achieved using gradient elution by mixing 2.00 M formic acid and Milli-Q water. There was still some peak overlapping, even with the optimized program. This problem was solved by setting the MS instrument in MRM mode. The typical chromatogram for the standard solution is shown in Figure 1. One can compete coelution of PHE and AGM. However, because the MS/MS system was used for detection, accurate quantification of the BAs was obtained by using separate MRM fragments for the individual BAs in order to overcome the main problem of coupling ion chromatography with mass spectrometry.

In our study, formic acid was used for the separation of 10 BAs most frequently found in the fishery products using a gradient elution described in Section 2. A good separation of nine BAs was obtained, including TYR, as seen in Figure 1.

It should be noted that AGM was co-eluted with PHE. This problem can be overcome by specific MRM detection, in which separation by *m/z* ratio results in complete “separation” of both peaks. In order to obtain complete chromatographic separation of all 10 BAs using the above-mentioned eluent, it would be necessary to substantially prolong the chromatographic run. So, in order to stay within reasonable time limits, we decided to keep the proposed chromatographic conditions, since the quantification of both BAs was still possible.

Validation of the described method was performed. The repeatability, reproducibility, linearity, and detection limits were determined. The reproducibility was determined at two concentration levels; that is, at 500 and 5000 ppb. A 12-point calibration curve was prepared for each BA, with concentrations ranging from 10 to 10,000 ng/mL. The detection limit was calculated as three times the standard deviation of the blank sample. The results are presented in Table 2.

The repeatability was expressed as the relative standard deviation (RSD) and was around 10% and 5% for almost all the BAs for the 500 bbp and 5000 ppb concentration levels, respectively. The worst results were obtained for SPM and SPMD because the late eluted peaks were more dispersed, so the determination of accurate peak area was hindered. The baseline noise for these peaks was larger as well. For most BAs, the linear response was in a wide concentration interval, for some even covering three orders of magnitude. It was also evident that for all amines, the Pearson’s correlation coefficient was higher than 0.99. Eight BAs showed relatively low detection limits, ranging from 20 ng/g of fish product up to around 400 ng/g. SPMD and SPM showed significantly higher detection limits. Both of them were eluted very late in the chromatogram, where peaks were already very dispersed due to the lower elution power of formic acid compared to methanesulfonic acid. The baseline noise level for the late eluted peaks was much higher as well.

Extraction recovery of the real samples was tested using Milli-Q distilled water and 1 M formic acid as a solvent. In general, the filtering was much slower for the samples in which acid was used for the elution compared to the ones with Milli-Q distilled water. Therefore, only Milli-Q distilled water was used for extraction for further studies.

**Figure 2:**
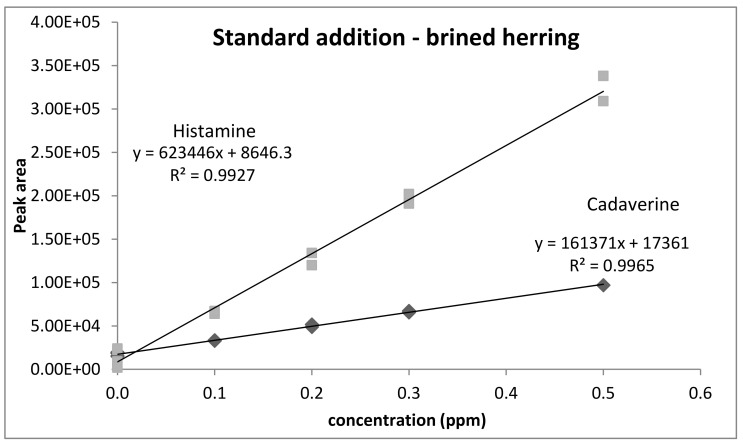
Determination of histamine and cadaverine by the standard addition technique.

Figure 2 shows the results for HIS and CAD determined by the standard addition technique. Note that concentration on the x-axis is concentration in the solution, therefore the concentration in fish was 10 times higher. Excellent linearity was observed with the standard addition technique. The method sensitivity was the same as for pure standards, so we can state that no matrix effect could be observed. The daily linear calibration curve using pure standards was therefore used for the evaluation of BA concentrations in the real samples.

**Figure 3:**
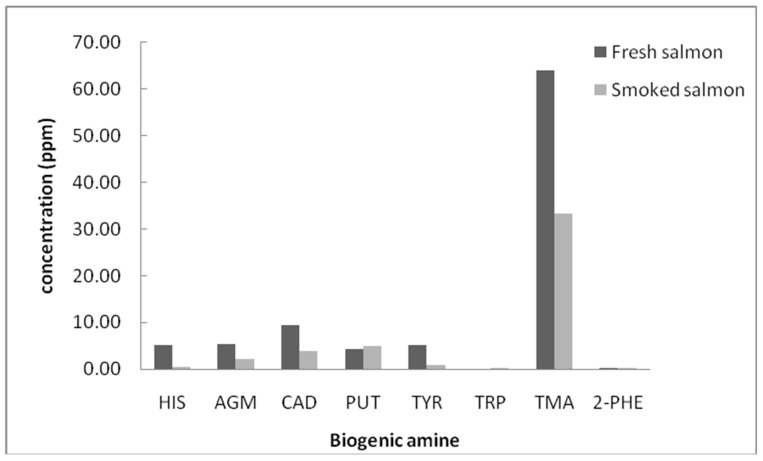
Concentrations of some biogenic amines in fresh and smoked salmon. HIS: Histamine, AGM: Agmatine, CAD: Cadaverine, PUT: Putrescine, TYR: Tyramine, TRP: Tryptamine, TMA: Trimethylamine, 2-PHE: 2-phenylethylamine

In Figure 3, the concentrations of BAs in fresh and smoked salmon are shown. It can be seen that in general, the concentrations of BAs in smoked salmon were lower than in fresh salmon samples. Both samples had the highest content of trimethylamine and the lowest content of TRP and PHE, whereas the contents of other BAs were comparable.

**Figure 4:**
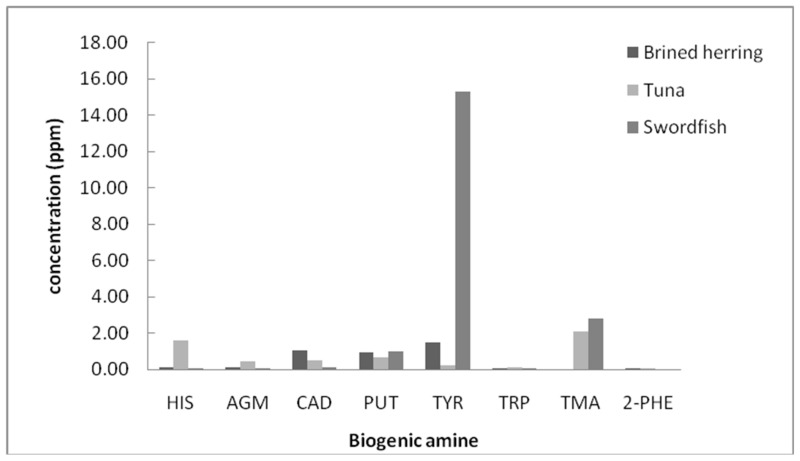
Concentrations of some biogenic amines in brined herring, fresh tuna, and swordfish. HIS: Histamine, AGM: Agmatine, CAD: Cadaverine, PUT: Putrescine, TYR: Tyramine, TRP: Tryptamine, TMA: Trimethylamine, 2-PHE: 2-phenylethylamine

In Figure 4, a comparison of the concentrations of BAs in brined herring, fresh tuna, and swordfish is shown. The concentrations of BAs in these samples were generally lower compared to the salmon samples shown in Figure 3. Apart from TMA, we also found elevated concentrations of TYR in the brined herring and swordfish samples.

**Figure 5:**
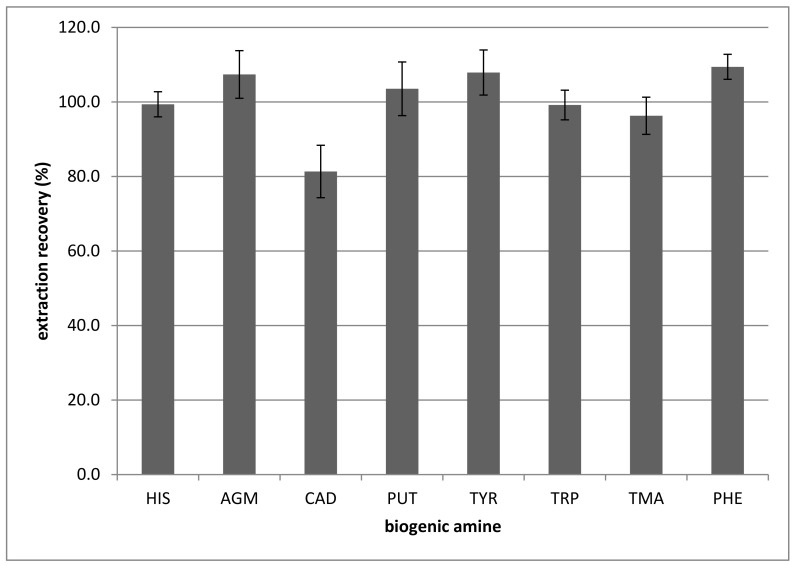
Extraction recoveries for some biogenic amines using the extraction method described in Section 2. HIS: Histamine, AGM: Agmatine, CAD: Cadaverine, PUT: Putrescine, TYR: Tyramine, TRP: Tryptamine, TMA: Trimethylamine, 2-PHE: 2-phenylethylamine.

The extraction recovery also was measured in fresh salmon; the results are presented in Figure 5. Generally, fresh salmon proved to be an extremely difficult sample concerning sample preparation. In all the samples, fresh salmon was the one from which filtered and homogenous extract was very difficult to prepare. The extraction recoveries were 100% at a 95% confidence interval, except for AGM and CAD. The error bars in Figure 5 show a 95% confidence interval. The extraction efficiencies for the AGM and CAD were significantly lower, at 35% and 17%, respectively.

The above-described analytical procedure was used for the determination of BAs in various traditional fish products obtained from seven different countries. The results for nine BAs are shown in Table 3. The reported results were corrected based on the above-mentioned extraction recoveries. The results for SPM were excluded, since the concentration levels were below the detection limit in all analyzed samples. The most abundant BAs were HIS, PUT, CAD, TYR, and AGM. The HIS level exceeded the permitted level of 100 ppm set by the EU and the authorities of most other countries in Turkish brined fish in herbal sauce. The product usually contains low salt levels and was obtained from a local producer. These producers keep any salted fish at ambient temperature, which accelerates HIS formation. Along with brined fish in herbal sauce, the fish paste of an Italian company contained 72.0 ppm HIS, which was over the permitted level of the FDA but still within acceptable quality according to EU authorities. The other Italian salted fish also was close to FDA limits. Much higher values were reported by the Rapid Alert System for Food and Feed (RASFF) portal [7] for the various fish products that originated from different countries. BA contents depend on various factors, as stated in Section 1. In particular, factors affecting the formation of these amines are much varied for traditionally processed fish in comparison with fresh fish products, as discussed in detail by Köse [6]. Fermented or similar enzymatically ripened fish products contain a higher non-protein nitrogen faction of free amino acids (FAA), which are the main precursors of BAs. Therefore, the FAA concentration in these products depends on the activity of endogenous enzymes, which in turn is favored by the denaturation of proteins as a consequence of acidity increase, dehydration, and the action of NaCl. The presence and the strains of amino acid decarboxylating bacteria can also vary in traditional fish products, particularly fermented fish, due to possibly used starter cultures. Moreover, high salt contents in fish products can decrease bacterial activity; therefore, BA formation will also be decreased. All these conditions contribute to a great extent to the variation of BAs in fish products [6]. Our earlier studies on the determination of BAs in fish products also showed a great variation in different traditional fish products from EU countries and Turkey [29,30].

Various outbreaks of HFP were reported by the EU in the RASFF portal, and many fish products were withdrawn from the market, or border rejection occurred. The detected HIS levels were highly variable in the analyzed lots [7]. Therefore, for low amounts of HIS and other BAs, it is suggested that seafood companies use hazard analysis critical control point (HACCP) plans during processing and marketing. FDA guidelines suggest using 17 ppm HIS for HACCP plans to avoid the HIS health risk. For an effective HACCP application in the seafood industry, rapid analytical techniques are required, since HIS and other BAs can continue to form even at a chilled temperature; therefore, the time to take action is very crucial. Currently, histamine test kits (mainly immunoassays) are used for such purposes. These commercial kits are usually obliged to be validated against an officially approved analytical method, either by the EU or FDA, in order to gain a buyer’s confidence. Various test kits have been validated against some reference methods to receive AOAC certification [31,32,33,34]. However, as discussed in Section 1, the past methods applied to fish and fishery products are lacking and have various drawbacks, mainly the necessary derivatization step, matrix effect, pre-column separation, and longer analytical time [6,17,22,23,24,25,26,27,28,35,36]. Stroka et al. [35] compared the reference methods of the Codex-approved fluorescence method (AOAC 977.13) and the EU HPLC method to analyze BAs in fish products. They reported that although the EU-mandated method was very accurate when applied to fresh tuna, a distinct matrix influence was noticed for all other fish species tested, leading to an overestimation of the histamine content. Duflos et al. [36] also pointed out a matrix effect during the analysis of BAs in some fish samples by the EU-accepted HPLC method that caused low recoveries. They further validated the method with some improvements. In a previous study, we also used the EU-mandated HPLC method to analyze these amines. The method was previously modified from Eerola et al. [37], and it was difficult to use an internal standard, as in the original procedure, since it interfered with unidentified peaks, which were also mentioned by Stroka et al. [35] and Duflos et al. [36]. We also observed various unknown peaks in the sample chromatograms of different traditional fish products that made us select a different internal standard to implement the current HPLC methods [29,30]. The newly modified method can overcome these problems easily, with the specific and sensitive identification of BAs at very low and high concentrations. Therefore, the currently developed IC-MS/MS method can offer a shorter analysis time to be used in HACCP plans, as well as be a candidate reference method for the future for validation purposes of quantitative and semi-quantitative test kits.

TMA is one of the main volatile amines produced by spoilage bacteria during fish spoilage, and is very often used as a food-quality parameter for fish and fishery products [16]. An upper limit of 10–15 mg TMA-N/100 g fish is usually suggested for the acceptability of fresh fish for human consumption [38]. Only one sample (fermented herring) showed higher levels of the suggested value. Other researchers also used various BAs for estimating the quality of fish and fishery products. For this purpose, two different formulae were suggested. The original formula was called the “Quality Index (QI)” by Mietz and Karmas [39]. The formulae used were as follows: QI = (HIS + PUT + CAD)/(1 + SPMD + SPM), which is also known as the Mietz and Karmas index [33]. The other was BAI = (HIS + PUT + CAD + TYR), which was suggested by Veciana-Nogues et al. [40]. According to these indexes, BAI or QI < 1 represents good quality, <1 and <10 indicates that the fish is in a state of alteration, and >10 indicates the state of spoilage/decomposition [41]. Therefore, identification of all amines in fish products will help to avoid food safety and to identify the products’ quality. Most recently developed LC-MS/MS methods were not applied in the determination of SPMD and SPM. Therefore, our study indicates the advantage of determining 10 BAs, including SPMD and SPM, all together in fish products in various fish matrices.

## 4. Conclusions

Mass spectrometry, especially in MRM mode, offers some important advantages over suppressed conductivity. It is much more selective since there are three parameters monitored, according to which we can confirm or reject the presence of the analyte: retention time, parent ion *m*/*z* ratio, and fragment ion *m*/*z* ratio. In addition, a relatively low limit of detection could be achieved, since by monitoring only one pair of ions for each analyte, all other background noises were filtered out.

Therefore, a robust and sensitive IC-MS/MS method was developed for the determination of 10 BAs in the fish products. The separation was achieved by using an MS-compatible solvent without reduction of the separation parameters. Out of these, a quantitative separation was achieved for nine of them. Although the linearity of the procedure was generally fine for all 10 BAs, two of them showed very high detection limits. Therefore, it can be concluded that the described procedure was suitable for the determination of eight BAs in the fish products. The most abundant BAs found in the real samples were HIS, PUT, CAD, TYR, and AGM. Their presence most likely can be attributed to the processing and storage conditions; however, detailed knowledge of the processing and storage parameters would be needed to address this issue.

## Figures and Tables

**Table 1:** Experimental conditions of the IC-MS/MS procedure.

**Eluent**	**Time**	**HCOOH (M)**
	−5	0.4
	0	0.4
	15	0.4
	25	2
	50	2
**Detection**	**MRM Transition**	**Collision Energy (V)**
Histamine (HIS)	112/95	20
Agmatine (AGM)	131/72	21
Cadaverine (CAD)	103/86	13
Putrescine (PUT)	89/72	13
Spermidine (SPMD)	146/72	21
Spermine (SPM)	203/84	41
	203/112	23
Tyramine (TYR)	138/121	15
	138/77	36
Tryptamine (TRP)	161/144	15
Trimethylamine (TMA)	60/45	21
2-Phenylethylamine (PHE)	122/105	20
	122/77	40

Note: the dwell time was 50 ms.

**Table 2:** Methods characteristics (linearity, R^2^, and limit of detection for each analyte).

Analyte	Linearity (mg L^−1^)	R^2^	LOD (ng/g)
TMA	0.03–2	0.9949	0.14
TYR	0.02–10	0.9999	0.02
PUT	0.2–10	0.9999	0.39
CAD	0.1–10	0.9993	0.20
HIS	0.03–10	0.9982	0.02
PHE	0.01–10	0.9993	0.02
AGM	0.2–10	0.9987	0.22
SPMD	1–10	0.9974	1.03
TRP	0.05–10	0.9997	0.04
SPM	5–10	0.9975	15.6

**Table 3:** The results of various traditional fish products for biogenic amine contents (ppm) analyzed by the newly developed IC-MS/MS method.

No	Sample Type	HIS	AGM	CAD	PUT	SPMD	TYR	TRP	TMA	2-PHE
1	Dry salted bonito, Turkey	23.4 ± 1.1	5.2 ± 1.3	3.5 ± 0.4	nd	2.8 ± 0.1	0.5 ± 0.1	nd	12.7 ± 4.7	nd
2	Dry salted herring, Turkey	3.1 ± 0.1	12.8 ± 1.4	18.8 ± 1.5	0.3 ± 0.5	nd	0.5 ± 0.1	nd	nd	nd
3	Brined anchovy, Turkey	nd	nd	nd	nd	nd	nd	nd	13.0 ± 1.4	nd
4	Brined anchovy, Turkey	1.5 ± 0.3	nd	nd	nd	nd	nd	nd	31.1 ± 1.3	0.8 ± 0.2
5	Brined bluefish, Turkey	14.1 ± 0.6	nd	8.4 ± 0.1	nd	nd	0.9 ± 0.1	nd	34.2 ± 4.7	n.d
6	Brined anchovy in herbal sauce, Turkey	226.9 ± 9.5	109.4 ± 6.4	142.5 ± 2.3	14.2 ± 2.7	5.8 ± 0.6	35.6 ± 10.1	nd	63.8 ± 17.8	1.0 ± 1.0
7	Brined bonito (*Lakerda* *), Turkey	0.4 ± 0.04	nd	nd	nd	nd	nd	nd	nd	nd
8	Turkish smoked salmon	0.3 ± 0.3	nd	nd	2.0 ± 0.3	3.0 ± 0.1	nd	nd	nd	nd
9	Italian brined anchovies in oil	44.7 ± 9.3	24.1 ± 6.6	25.8 ± 5.9	4.8 ± 1.12	n.d	7.6 ± 1.7	n.d	24.4 ± 4.9	0.8 ± 0.2
10	Italian fish paste	72.3 ± 1.2	14.5 ± 1.2	12.4 ± 0.5	5.5 ± 0.4	3.2 ± 0.4	6.4 ± 0.4	177.2 ± 1.9	77.6 ± 4.1	4.5 ± 0.2
11	Salted anchovy, Slovenia	12.0 ± 0.8	41.3 ± 2.7	23.4 ± 2.4	5.5 ± 0.6	12.7 ± 0.4	14.8 ± 0.8	nd	64.1 ± 17.1	1.3 ± 0.1
12	Danish fermented herring	12.4 ± 0.4	52.0 ± 0.1	61.1 ± 1.1	35.7 ± 1.0	5.6 ± 0.9	33.5 ± 1.5	nd	270.6 ± 3.1	nd
13	Salted anchovy in oil, Greece	8.7 ± 0.4	138.6 ± 0.4	66.8 ± 5.1	19.0 ± 1.3	9.8 ± 0.2	50.2 ± 3.5	nd	82.4 ± 7.3	4.1 ± 0.2
14	Canadian smoked salmon	nd	nd	nd	10.4 ± 3.4	5.4 ± 2.5	77.2 ± 21.6	nd	9.6 ± 3.2	nd
15	Norwegian smoked salmon	nd	nd	nd	5.2 ± 2.1	nd	195.1 ± 2.6	nd	16.9 ± 5.5	nd
16	Norwegian marinated herring	nd	nd	nd	6.4 ± 1.9	5.0 ± 0.9	97.7 ± 22.3	nd	9.1 ± 3.7	nd
17	Norwegian smoked salmon	nd	nd	nd	5.9 ± 0.5	5.2 ± 0.6	178.1 ± 23.0	nd	8.2 ± 0.5	0.4 ± 0.4

Note: ± is SD of three determinations; nd: not detected, meaning the levels were below the detection limit of the method for the relating amine. TMA: trimethylamine, PUT: putrescine, CAD: cadaverine, HIS: histamine, AGM: agmatine, 2-PHE: 2-phenylethylamine, SPMD: spermidine, TRP: tryptamine. * Lakerda is a special brined product of Turkey and Greece (suspected of being slightly fermented).

## Data Availability

Not applicable.
